# Clinical and Immunological Characteristics of Patients With Adenovirus Infection at Different Altitude Areas in Tibet, China

**DOI:** 10.3389/fcimb.2021.739429

**Published:** 2021-10-13

**Authors:** Bowen Wang, Mengjia Peng, Li Yang, Guokai Li, Jie Yang, Ciren Yundan, Xiaohua Zeng, Qianqi Wei, Qi Han, Chang Liu, Ke Ding, Kaige Peng, Wen Kang

**Affiliations:** ^1^ Department of Prevention and Control of Infectious Diseases, Center for Disease Control and Prevention (CDC) of Tibet Military Command, Lhasa, China; ^2^ Department of Emergency, General Hospital of Tibet Military Command, Lhasa, China; ^3^ Department of Radiology, General Hospital of Tibet Military Command, Lhasa, China; ^4^ Department of Thoracic Surgery, General Hospital of Tibet Military Command, Lhasa, China; ^5^ Department of Infectious Diseases, General Hospital of Tibet Military Command, Lhasa, China; ^6^ Department of Laboratory, 954 Hospital of Army, Lhoka, China; ^7^ Department of Laboratory, 956 Hospital of Army, Nyingchi, China; ^8^ Department of Radiology, Xuchang People’s Hospital, Xuchang, China; ^9^ Department of Infectious Diseases, Tangdu Hospital, The Airforce Medical University, Xi’an, China

**Keywords:** altitude, adenovirus, clinic, immunology, epidemiology, character

## Abstract

**Background:**

The severities of human adenovirus (HAdV) infection are diverse in different areas of Tibet, China, where a large altitude span emerges. Serious consequences may be caused by medical staff if the clinical stages and immunological conditions of patients in high-altitude areas are misjudged. However, the clinical symptoms, immunological characteristics, and environmental factors of HAdV infection patients at different altitude areas have not been well described.

**Methods:**

In this retrospective, multicenter cohort study, we analyzed the data of patients who were confirmed HAdV infection by PCR tests in the General Hospital of Tibet Military Command or CDC (the Center for Disease Control and Prevention) of Tibet Military Command from January 1, 2019, to December 31, 2020. Demographic, clinical, laboratory, radiological, and epidemiological data were collected from medical records system and compared among different altitude areas. The inflammatory cytokines as well as the subsets of monocytes and regulatory T cells of patients were also obtained and analyzed in this study.

**Results:**

Six hundred eighty-six patients had been identified by laboratory-confirmed HAdV infection, including the low-altitude group (*n* = 62), medium-altitude group (*n* = 206), high-altitude group (*n* = 230), and ultra-high-altitude group (*n* = 188). Referring to the environmental factors regression analysis, altitude and relative humidity were tightly associated with the number of infected patients (*P* < 0.01). A higher incidence rate of general pneumonia (45.7%) or severe pneumonia (8.0%) occurred in the ultra-high-altitude group (*P* < 0.05). The incubation period, serial interval, course of the disease, and PCR-positive duration were prolonged to various extents compared with the low-altitude group (*P* < 0.05). Different from those in low-altitude areas, the levels of IL-1β, IL-2, IL-4, IL-6, IL-8, IL-10, G-CSF, GM-CSF, IFN-γ, IP-10, MCP-1, TNF-α, TNF-β, and VEGF in the plasma of the ultra-high-altitude group were increased (*P* < 0.05), while the proportion of non-classical monocytes and regulatory T cells was decreased (*P* < 0.05).

**Conclusions:**

The findings of this research indicated that patients with HAdV infection in high-altitude areas had severe clinical symptoms and a prolonged course of disease. During clinical works, much more attention should be paid to observe the changes in their immunological conditions. Quarantine of patients in high-altitude areas should be appropriately extended to block virus shedding.

## Introduction

Since the discovery and isolation of adenovirus in the 1950s, more than 100 serotypes had been identified. There are 52 kinds of human adenoviruses (HAdVs), which could be divided into six subgroups: A, B, C, D, E, and F (Greber and Flatt, 2019). HAdVs are able to infect the human respiratory tract, gastrointestinal tract, urethra, bladder, eye, and liver. The typical symptoms of respiratory tract infection caused by HAdVs are cough, nasal obstruction, and pharyngitis, accompanied by fever, chills, headache, and muscle pain at the same time (Ison and Hirsch, 2019). Sporadic cases and outbreaks of HAdVs have been recorded in both USA and China, with cases totaling in hundreds, especially for children and military recruits. Fortunately, mortality has been estimated at a relatively low level (Gautret et al., 2014). It had been reported that HAdV pneumonia accounted for about 10% of childhood pneumonia, which was mostly caused by HAdV types 3 and 7 (Zou et al., 2021). In adolescence, the mortality of HAdV pneumonia is 8%–10% (Scott et al., 2016; Kujawski et al., 2020). Notably, due to the highly infectious and rapid spreading of HAdVs, military recruits are a special group at high risk during endemic outbreaks (Vento et al., 2011; Hang et al., 2020). In 2006, HAdV-14 caused abrupt epidemics among US military trainees in five states at least: California, Georgia, Illinois, Montana, and Texas (Tate et al., 2009). In 2011, 43 children, attending primary school beside an air force military center, were proved to be infected by HAdV-14 in the Tongwei County of Gansu, China (Huang et al., 2013). Therefore, military hospitals and CDC have always been paying particular attention to the control and prevention of HAdVs for fear of virus shedding.

Studies had reported that the chance of residents suffering from respiratory diseases in high-altitude areas was more likely to be increased. Vargas et al. found that the incidence rate of asthma was higher if the residents lived above 1,500 m (Vargas et al., 2018). Khan et al., through a long-term cohort study of 5,204 children in Pakistan, confirmed that the risk of pneumonia in children was closely related to the altitude they lived (Khan et al., 2009). In addition, during the global outbreak of new coronavirus pneumonia in 2019, higher altitude usually indicates more severe clinical symptoms and worse outcomes in patients (Zeng et al., 2020; Woolcott and Bergman, 2020). Tibet, located in the southwest of China, is impressed by the broad area, complex terrain and vast altitude span. For example, the altitude of Pali County of Shigatse is more than 5,000 m, while Motuo County of Nyingchi is not enough to even 500 m (Ni et al., 2021). However, there have not been reports on the clinical and immunological characteristics of HAdV infection in different areas of Tibet.

Although most HAdV infectious patients have the respiratory disease with mild or asymptomatic clinical manifestations, multisystem illnesses are also reported occasionally (Ison and Hirsch, 2019). Since lung physiology is heavily affected by the atmospheric pressure that decreases with altitudes, it is generally believed that respiratory diseases might be more serious in high-altitude areas (Truog et al., 2020). However, many medical staff still might not have a clear idea about the state of patients in high-altitude areas, without the preparations for the rapid changes of illness or the prolonged course of disease and incubation period. Especially for referral patients from high-altitude areas, medical staff in low-attitude areas are more likely to misjudge their clinical stage, miss the best opportunity for treatment, and even result in serious HAdV outbreaks in the local areas. This study aims to reveal the epidemiological, clinical, laboratory, radiological, and immunological characteristics of patients with HAdV infection as well as the environmental risk factors in different altitude areas, to provide scientific proof for the diagnosis and treatment of HAdV infections in high-altitude areas.

## Methods

### Study Design and Participation

This retrospective, multicenter cohort study included four cohorts of patients from the General Hospital of Tibet Military Command in Lhasa, 954 Hospital of Army in Lhoka, 956 Hospital of Army in Nyingchi, and CDC of Tibet Military Command in Lhasa. All of the patients with confirmed HAdV infection who were admitted to the General Hospital, 954 Hospital, and 956 Hospital, as well as who were reported to CDC, were retrospectively enrolled from January 1, 2019, to December 31, 2020. This study was approved by the Research Ethics Commission of the General Hospital and the requirement for informed consent was waived by the Ethics Commission.

### Data Collection

Epidemiological data were collected through interviews and field investigation. Environmental data were obtained in CDC of Tibet Military Command. Demographic, clinical, laboratory, and radiologic data were extracted from electronic medical records by a standard data collection form that was a modification of the World Health Organization (WHO)/International Severe Acute Respiratory record form (Zhou et al., 2020). All data above were verified by two physicians and a third researcher adjudicated any differences in interpretations between them.

### Epidemiological Data Collection

The number of acute respiratory tract infections in military units of Tibet was reported to the CDC every month since January 1, 2019. Once clusters of cases emerged or suspected individuals were identified, the epidemiology team with members from the CDC of Tibet Military Command would be informed to initiate detailed field investigations and collect specimens including swabs, blood, stools, and urines at the acute phase for further tests.

Information about the date of illness onset, clinical symptoms, laboratory results, and the date of cure were collected through detailed interviews with infected persons, close contacts, and medical staff. Epidemiological data were acquired through interviews and field investigations. If necessary, the investigators interviewed each infected patient to verify the contact history within 2 weeks before the onset of the illness. Information about contact with other people with similar symptoms was also included. All of the data collected during the field survey, including contact history, the timeline of events, and identification of close contacts, had been cross-checked from multiple sources. The data were entered into the central database of the CDC and verified by EpiData Association.

### Environmental Data Collection

In a previous study, high altitude was defined as a geographic elevation of ≥1,500 m (Woolcott et al., 2015). Here, altitude was divided into four categories: 0–1,500, 1,500–3,000, 3,000–4,500, and ≥4,500 m. The average altitude of each region in Tibet was obtained from the CDC of Tibet Military Command and verified by Google Earth. All of the study groups were divided based on the altitude of the residence of patients. Besides, the average temperature, relative humidity, and oxygen content of each month in different areas of Tibet were acquired from the local CDC.

### Clinical Data Collection

The recent exposure history, clinical symptoms or signs, and results of laboratory tests on admission of inpatients and outpatients were obtained from the hospital electronic medical records. These data of the non-referral patients, if they had, were collected from the local medical systems. Radiologic evaluation including chest X-ray or computed tomography (CT), as well as laboratory testing, was performed according to the clinical needs of patients. Referring to the documents or descriptions in the medical record system, we determined whether the patient had radiologic abnormalities; if image scanning can be carried out, the attending physician of the respiratory department would review and extract the data. Disagreements between the two reviewers were resolved through negotiation with a third reviewer. Laboratory evaluation consisted of whole blood count, blood chemistry analysis, coagulation test, liver and kidney function evaluation, and measurement of electrolyte, C-reactive protein, procalcitonin, lactate dehydrogenase, and creatine kinase. Treatment data consisted of antiviral therapy, antibiotic therapy, use of corticosteroid, nasal cannula, non-invasive ventilation, and invasive mechanical ventilation. Prognosis of the infection could be divided into hospitalization, recovery, and discharge from hospital at the end of observation. All of the data mentioned above were entered into a computerized database and cross-checked in the CDC of Tibet Military Command. If the core data were lost, a request would be sent to the coordinator, who subsequently contacted the attending physician for further clarifications.

### Laboratory Procedures

The nasopharyngeal swabs, blood, and fecal specimens during the acute phase of HAdV infection were subsequently sent to the central laboratory of the General Hospital or CDC for etiological detection. Blood samples were centrifuged at 500×*g* and the upper plasma was collected for following nucleic acid extraction experiments. Each 1 g fecal sample was added to 10 ml of 0.9% normal saline. Then, the samples were centrifuged at 500×*g* and the supernatant was collected for the following nucleic acid extracting tests. Nucleic acids of pathogens were extracted from different specimens using a QIAamp Viral RNA Mini Kit (Qiagen) according to the recommended protocol of the manufacturer. A diagnostic kit for HAdVs (Beijing Kinghawk) was applied for HAdV nucleic acid detection referring to the recommended protocol of the manufacturer. Briefly, the presence of HAdV nucleic acids in samples was detected by real-time PCR. The primer and probe sequences of the target were listed as follows: forward primer 5′-GCCACGGTGGGGTT TCTAAACTT-3′, reverse primer 5′-GCCCCAGTGGTCTTAC ATGCACATG-3′, and the probe 5′-FAM-TGCACCAGAC CCGGGCTCAGGTACTCCGA-3′-BHQ. Conditions for amplification were 95°C for 5 min, followed by 40 cycles of 95°C for 10 s and 58°C for 40 s. A cycle threshold value (Ct-value) of each specimen less than 37 was defined as a positive test, while a Ct-value of 40 or more was defined as a negative test. A medium viral load with a Ct-value of 37 to 40 required confirmation by retesting.

### Case Definitions

A confirmed case of HAdV infection was defined as that whose respiratory, blood, or fecal specimens were positive for HAdVs by real-time PCR. A new identified case was in contact with or epidemiological association with a confirmed patient within 14 days before the onset of the disease. The definition of pneumonia cases with HAdV infection was based on the definition of community-acquired pneumonia cases recommended by the WHO (Metlay et al., 2019): fever, with or without temperature records; radiographic evidence of pneumonia; low or normal white blood cell count or low lymphocyte count; no remission of symptoms after 3 days of antimicrobial treatment; and etiological evidence. Diagnosis for severe pneumonia with HAdV infection also followed the WHO criteria (Jain et al., 2015): one primary criterion or more than three secondary criteria were met. Primary criteria were mechanical ventilation with tracheal intubation or vasoactive drugs required for septic shock after active fluid resuscitation. Secondary criteria were respiratory rate ≥30 times/min, oxygenation index ≤250 mmHg, multiple lobar infiltrations, disturbance of consciousness or disorientation, blood urea nitrogen ≥7.14 mmol/L, systolic blood pressure <90 mmHg, and requiring active fluid resuscitation.

### Cytokine and Chemokine Measurement

The plasma cytokines and chemokines, interleukin (IL)-1β, IL-2, IL-4, IL-6, IL-8 (also known as CXCL8), IL-10, IL-12, IL-13, IL-15, granulocyte colony-stimulating factor (G-CSF, also known as CSF3), granulocyte–macrophage colony-stimulating factor (GM-CSF, also known as CSF2), interferon γ (IFN-γ), induced protein 10 (IP-10, also known as CXCL10), MCP-1 (also known as CCL2), monocyte chemoattractant protein 1 (MIP-1, also known as CCL3), tumor necrosis factor α (TNF-α), tumor necrosis factor β (TNF-β), and vascular endothelial growth factor (VEGF), were measured in the acute phase of illness. According to the instructions of the manufacturer, the inflammatory factors and chemokines of all patients were detected using ELISA kit (R&D System). The optical density (OD) value of the experimental results was read on a microplate reader (Bio-Rad).

### Flow Cytometry

The blood samples during the acute phase of HAdV infection were collected and sent to the central laboratory of the General Hospital or CDC for flow cytometry. Total peripheral blood mononuclear cells (PBMCs) were separated from blood samples in the acute phase of illness. Then, PBMCs were stained with conjugated antibodies against the following proteins: CD45-PerCp (2D1), CD4-PE/Cy7 (OKT4), CD14-FITC (63D3), CD16-APC (3G8), CCR2-APC/Cy7 (K036C2), CX3CR1-PE/Cy7 (2A9-1), and CD86-PE (BU63) (all from Biolegend). While the intracellular detection of Foxp3 with Foxp3-PE (295D) (Biolegend) was performed on fixed and permeabilized cells using FOXP3 Fixation/Permeabilization Buffer (Biolegend) according to the instructions of the manufacturer. The lymphocyte and monocyte gates were both based on CD45. Regulatory T cells (Tregs) were defined as CD4^+^ and Foxp3^+^ among lymphocytes. The subsets of monocytes were recognized by both CD14 and CD16 (Cros et al., 2010). In addition, the expression of CCR2, CX3CR1, and CD86 was determined in different subsets of monocytes, respectively.

### Statistics

The epidemic curve of HAdV and acute respiratory tract infections in Tibet was drawn according to the number of incident cases and month of diagnosis. Sensitivity analysis was also carried out to include the important outbreak events in the epidemic curve. In the infectious population, multilevel mixed-effect Poisson regression analysis was applied to estimate the correlation of altitude, temperature, relative humidity, and oxygen content with the number of patients. The incubation period distribution (the time delay from infection to illness onset), course of disease, and PCR-positive duration were estimated by fitting the log-normal distribution to the data of exposure history and onset date. The serial interval (the delay between illness onset dates in successive cases in a transmission chain) is estimated by fitting the gamma distribution of cluster survey data. Continuous variables and categorical variables were expressed as median (IQR) and *n* (%), respectively. We applied the Kruskal–Wallis test (followed by *post-hoc* analysis with Dunnett’s *t*-test with Bonferroni adjustment when appropriate) to compare the continuous variables among different altitudes, while the *χ* ^2^ test or Fisher exact test was employed for the comparison of categorical variables. The Kruskal–Wallis test was also used to compare the changes of leukocyte count, lymphocyte count, platelet count, D-dimer, urea nitrogen, and creatinine at different days after disease onset. Dunnett’s *t*-test was used to compare plasma inflammatory factors, Tregs, and monocyte subsets between the middle-altitude group, high-altitude group, ultra-high-altitude group, and low-altitude group. Statistical analyses were performed using SPSS Version 22.0. Two-tailed *P*-values less than 0.05 were considered statistically significant unless there are special explanations.

## Results

### Epidemic Curve and Environmental Factors of HAdV Patients in Tibet

There were 70.63% of acute respiratory tract infections in Tibet that concentrated from September to March of next year (**Supplementary Figure 1**). The epidemic curve of HAdV infection suggested that the number of cases reached a peak in December and January (**Supplementary Figure 1**). However, epidemic curves of HAdV infection and respiratory diseases did not coincide completely due to a small fluctuation of HAdV cases in April and May. Despite gathering in spring and winter, some sporadic HAdV cases also emerged all around the year. The majority of the HAdV outbreaks occurred in Lhasa, accounting for 50% of the total incidents. Field investigations demonstrated that HAdV infections might spread across the areas due to the mobility of patients.

**Figure 1:**
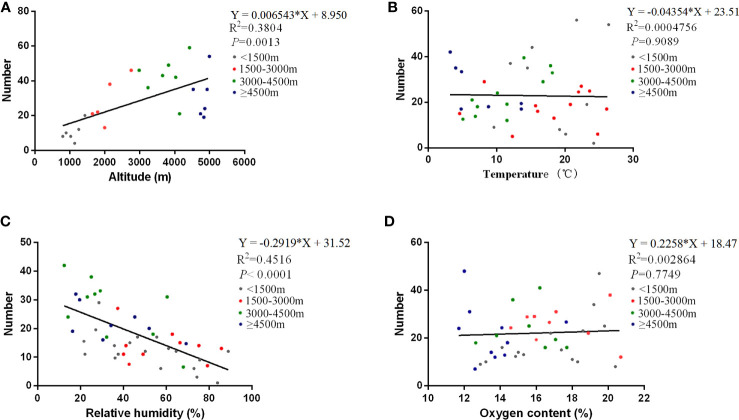
Correlations of the number of patients with various environmental factors. The correlations of the number of HAdV infections with various environmental factors are shown in altitude **(A)**, temperature **(B)**, relative humidity **(C)**, and oxygen content **(D)**, respectively. The curves in the figure are derived from the Poisson regression model. The equation, correlation coefficient, and *P*-value of the curves are marked at the top right of every image.

Generally speaking, environmental factors have vital roles in the onset, transmission, and maintenance of HAdV infections (Gautret et al., 2014; Yao et al., 2021). Here, we took the four environmental factors involving the altitude of residence of the patients, temperature, relative humidity, and oxygen content into account (**Figure 1**). Different from the traditional concepts, the temperature and oxygen content were not related to the number of HAdV patients (*P* > 0.05). However, altitude and relative humidity were the environmental factors affecting the number of patients (*P* < 0.01). Furthermore, altitude was positively correlated with the number of patients (**Figure 1A**), while a negative correlation was recognized between relative humidity and the number of patients (**Figure 1C**).

### Demographic, Clinical, and Epidemiological Characteristics of HAdV Patients at Different Altitude Areas

Six hundred eighty-six patients had been identified by laboratory-confirmed HAdV infection, consisting of the low-altitude group (*n* = 62), medium-altitude group (*n* = 206), high-altitude group (*n* = 230), and ultra-high-altitude group (*n* = 188). The demographic and clinical characteristics of the patients are shown in **Table 1**. The median age of all patients was 26 years (interquartile range, 19–32), and 93.4% of the patients were male. There was no significant difference in age and gender among the groups (*P* > 0.05). A total of 2.6% of the patients were medical staff, and 38.9% of them were in the ultra-high-altitude group (**Table 1**).

**Table 1:** Demographics and clinical characteristics of study patients.

	Number (%)					*P*-value		
	Total (*n* = 686)	Low-altitude group (<1,500 m) (*n* = 62)	Medium-altitude group (1,500–3,000 m) (*n* = 206)	High-altitude group (3,000–4,500 m) (*n* = 230)	Ultra-high-altitude group (≥4,500 m) (*n* = 188)	*P*_1_	*P*_2_	*P*_3_
**Age (IQR)**	26 (19–32)	26 (21–30)	25 (19–30)	25 (19–31)	27 (20–33)	0.521	0.534	0.462
**Sex**								
Male	641 (93.4)	58 (93.5)	192 (93.2)	214 (93.0)	177 (94.1)	>0.99	>0.99	0.68
Female	45 (6.6)	4 (6.5)	14 (6.8)	16 (7.0)	11 (5.9)			
**Infected**								
Patients	668 (97.4)	61 (98.4)	201 (97.6)	225 (97.8)	181 (96.3)	0.58	0.63	0.37
Medical staff	18 (2.6)	1 (1.6)	5 (2.4)	5 (2.2)	7 (3.7)			
**Current smoking**	453 (66.0)	32 (51.6)	115 (55.8)	162 (70.4)	145 (76.6)	0.56	0.007	<0.001
**Comorbidity**								
Hypertension	37 (5.4)	3 (4.8)	8 (3.9)	13 (5.7)	13 (6.9)	0.72	>0.99	0.77
Cardiovascular disease	12 (1.7)	1 (1.6)	3 (1.5)	3 (1.3)	5 (2.7)	>0.99	>0.99	>0.99
Diabetes	18 (2.6)	2 (3.2)	5 (2.4)	6 (2.6)	5 (2.7)	0.66	0.68	>0.99
Cerebrovascular diseases	9 (1.3)	0	2 (1.0)	4 (1.7)	3 (1.6)	>0.99	0.58	>0.99
COPD	32 (4.7)	2 (3.2)	6 (2.9)	9 (3.9)	15 (8.0)	>0.99	>0.99	0.26
Chronic kidney disease	8 (1.2)	0	1 (0.5)	2 (0.9)	5 (2.7)	>0.99	>0.99	>0.99
Chronic liver disease	48 (7.0)	2 (3.2)	9 (4.4)	13 (5.7)	24 (12.8)	>0.99	0.75	0.03
Other	32 (4.7)	5 (8.1)	6 (2.9)	9 (3.9)	12 (6.4)	0.13	0.19	0.77
**Signs and symptoms**								
Fever	312 (45.5)	22 (38.7)	65 (31.6)	116 (50.4)	109 (58.0)	0.64	0.045	0.003
Fatigue	264 (38.5)	8 (12.9)	34 (16.5)	107 (46.5)	115 (61.2)	0.56	<0.001	<0.001
Cough	531 (77.4)	39 (62.9)	152 (73.8)	178 (77.4)	162 (86.2)	0.11	0.032	<0.001
Sputum production	209 (30.5)	10 (16.1)	28 (13.6)	74 (32.2)	97 (51.6)	0.68	0.037	<0.001
Shortness of breath	137 (20.0)	5 (8.1)	19 (9.2)	54 (23.5)	59 (31.4)	>0.99	0.007	<0.001
Sore throat	128 (18.7)	5 (8.1)	14 (6.8)	48 (20.9)	61 (32.4)	0.78	0.025	<0.001
Abdominal pain	42 (6.1)	3 (4.8)	10 (4.9)	18 (7.8)	11 (5.9)	>0.99	0.083	>0.99
Diarrhea	35 (5.1)	3 (4.8)	8 (3.9)	14 (6.1)	10 (5.3)	0.73	0.277	>0.99
Nausea	38 (5.6)	4 (6.5)	8 (3.9)	15 (6.5)	11 (5.9)	0.48	>0.99	>0.99
Vomiting	26 (3.8)	2 (3.2)	6 (2.9)	10 (4.3)	8 (4.3)	>0.99	>0.99	>0.99
Respiratory rate (IQR)	23 (20–27)	20 (19–22)	22 (20–24)	24 (21–26)	25 (22–29)	0.75	0.043	0.014
Heart rate (IQR), bpm	86 (77–97)	80 (73–88)	85 (79–92)	89 (80–101)	92 (82–103)	0.83	0.004	<0.001
Mean arterial pressure (IQR), mmHg	88 (81–96)	85 (78–91)	86 (81–92)	91 (83–100)	95 (88–104)	>0.99	0.036	<0.001
**Pneumonia**								
General	214 (31.2)	10 (16.1)	47 (22.8)	71 (30.9)	86 (45.7)	0.29	0.025	<0.001
Severe	29 (4.2)	0	2 (1.0)	12 (5.2)	15 (8.0)	>0.99	0.077	0.026
**Treatment**								
Antiviral therapy	628 (91.5)	48 (77.4)	192 (93.2)	218 (94.8)	170 (90.4)	0.001	<0.001	0.014
Antibiotic therapy	665 (96.9)	58 (93.5)	202 (98.1)	221 (96.1)	184 (97.9)	0.086	0.49	0.11
Use of corticosteroid	223 (32.5)	8 (12.9)	45 (21.8)	78 (33.9)	92 (48.9)	0.15	<0.001	<0.001
Nasal cannula	378 (55.1)	30 (48.4)	117 (56.8)	120 (52.2)	111 (59.0)	0.25	0.67	0.18
Non-invasive ventilation	172 (25.1)	12 (19.4)	41 (19.9)	62 (27.0)	57 (30.3)	>0.99	0.25	0.10
Invasive mechanical ventilation	26 (3.8)	0	2 (1.0)	11 (4.8)	13 (6.9)	>0.99	0.13	0.042
**Prognosis**								
Hospitalization	19 (2.8)	0	1 (0.5)	8 (3.5)	10 (5.3)	0.84	0.16	0.026
Recovery	593 (86.4)	58 (93.5)	190 (92.2)	196 (85.2)	149 (79.3)	–	–	–
Discharge from hospital	74 (10.8)	4 (6.5)	15 (7.3)	26 (11.3)	29 (15.4)	–	–	–

Data are median (IQR), n (%), or n/N (%), where N is the total number of patients with available data. P-values comparing among different altitude areas are from χ^2^ test, Fisher’s exact test, or Kruskal–Wallis test (followed by post-hoc analysis with Dunnett’s t-test with Bonferroni adjustment). P_1_ refers to the comparisons between the low-altitude group and medium-altitude group. P_2_ refers to the comparisons between the low-altitude group and high-altitude group. P_3_ refers to the comparisons between the low-altitude group and ultra-high-altitude group.

IQR, interquartile range; COPD, chronic obstructive pulmonary disease.

Current smoking accounted for 66.0% of all patients with HAdV infection (**Table 1**). Compared with the low-altitude group (51.6%), the proportion of current smoking in the high-altitude group (70.4%) and the ultra-high-altitude group (76.6%) was significantly higher (*P* < 0.01). Among all the clinical symptoms, the most common were cough (77.4%), fever (45.5%), fatigue (38.5%), and sputum (30.5%), and these symptoms seemed to be more obvious in the high-altitude group and ultra-high-altitude group (*P* < 0.05). Other relatively rare symptoms, such as shortness of breath (20.0%) and sore throat (18.7%), also increased in both high-altitude areas and ultra-high-altitude areas (*P* < 0.05). In addition, there were significant differences in respiratory rate, heart rate, and mean arterial pressure with the comparison of the high-altitude group and ultra-high-altitude group to the low-altitude group (*P* < 0.05). Notably, the incidence of general pneumonia (45.7%) and severe pneumonia (8.0%) in the ultra-high-altitude group was significantly higher than that in the low-altitude group (*P* < 0.05). Similar to the severity of disease, patients in ultra-high-altitude areas had a higher percentage in receiving antiviral therapy (90.4%), a corticosteroid to restrict inflammation (48.9%), and invasive mechanical ventilation (6.9%) (*P* < 0.05). Although the majority of patients recovered after treatment, patients at ultra-high-altitude areas showed a higher hospitalization rate (5.3%) while a lower recovery rate (79.3%) at the end of observation (*P* < 0.05).

**Figure 2:**
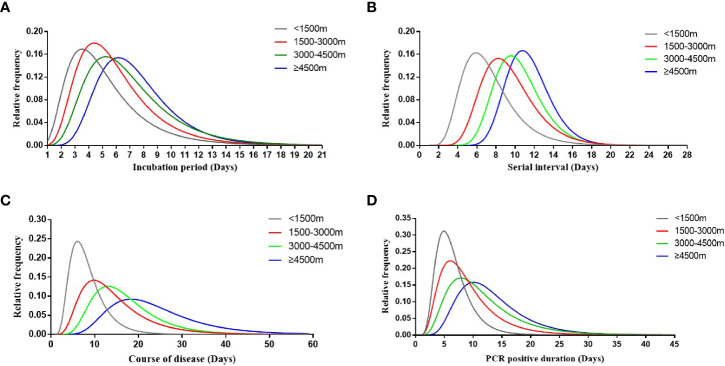
Key time-to-event distributions. The estimated incubation period distribution (i.e., the time from infection to illness onset) is shown in **(A)**. The estimated serial interval distribution (i.e., the time from illness onset in successive cases in a transmission chain) is shown in **(B)**. The estimated course of disease (i.e., the time from illness onset to the disappearance of symptoms) is shown in **(C)**. The estimated PCR-positive duration (i.e., the time from the first PCR positive to PCR negative) is shown in **(D)**.

Referring to field investigation of clusters of cases, we found that the epidemiological characteristics of HAdV patients at different altitude areas were far from the same (**Figure 2**). By statistical estimation, the median incubation period of the low-altitude group (3.5 days, 95% CI 2.7–4.3), middle-altitude group (4.4 days, 95% CI 4.2–4.6), high-altitude group (5.2 days, 95% CI 4.9–5.5), and ultra-high-altitude group (6.2 days, 95% CI 5.5–6.8) were described. In addition, 95% of the incubation period distribution in the low-altitude group was 11.3 days (95% CI 8.9–16.1), while that in the ultra-high-altitude group was 14.9 days (95% CI 10.3–21.5) (**Figure 2A**). Similarly, serial interval (**Figure 2B**), course of the disease (**Figure 2C**), and PCR-positive duration (**Figure 2D**) of HAdV patients also increased with altitude. For the duration of disease, the difference between the groups was particularly huge (**Figure 2C**). The median duration of disease in the low-altitude group was 6.1 days (95% CI 5.8–6.3), while that in the ultra-high-altitude group was significantly prolonged (18.3 days, 95% CI 17.2–19.4).

### Cytokines and Immunocytes of HAdV Patients at Different Altitude Areas

**Figure 3:**
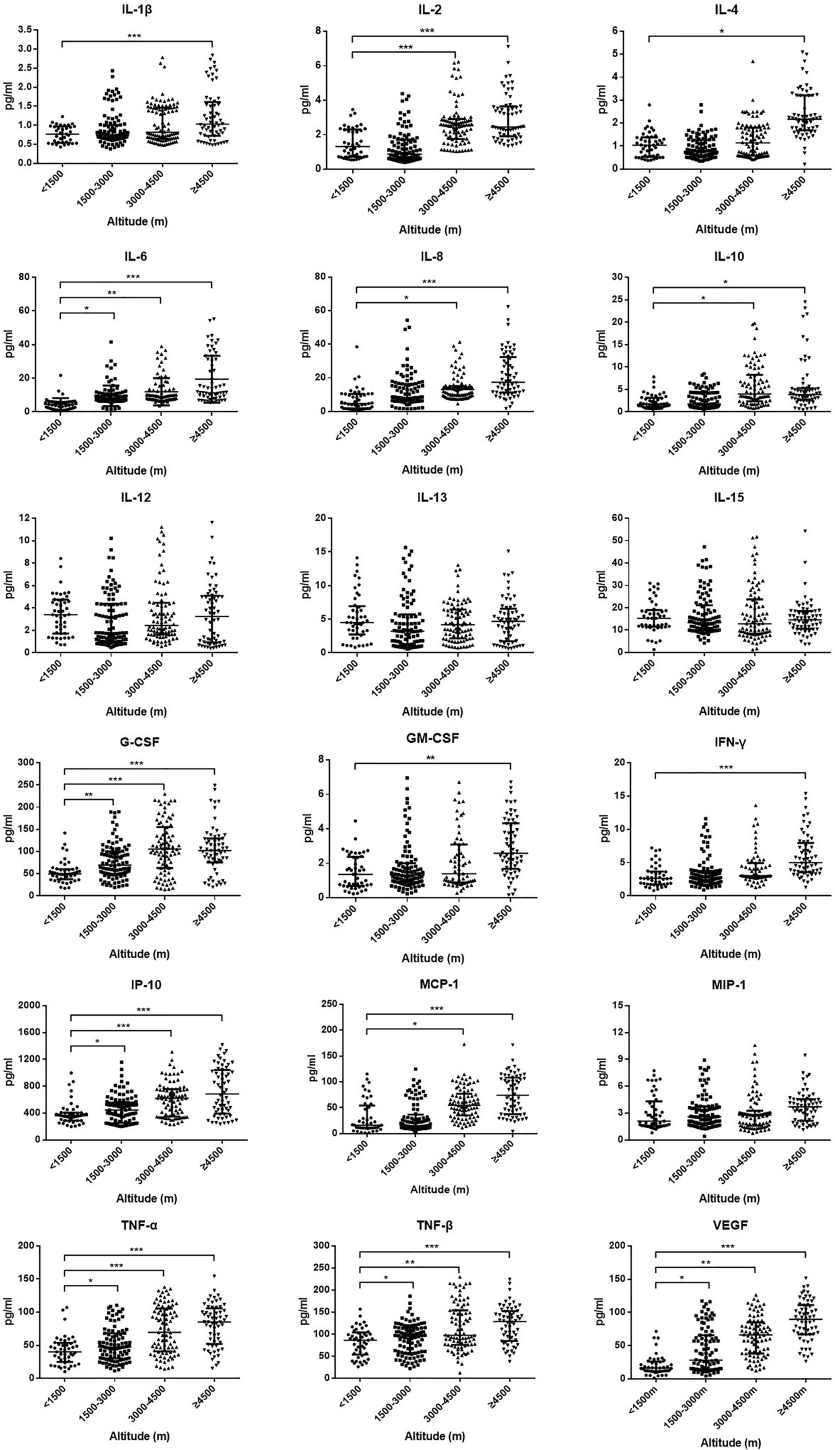
Plasma level of cytokines and chemokines among HAdV infectious patients at different altitudes. Comparison of cytokine and chemokine levels among HAdV infectious patients at different altitudes. The vertical axes differ for each of the mediators to accommodate the extreme variation in the normal distributed range of cytokine concentrations. Data represent median and interquartile range. The Kruskal–Wallis test (followed by *post-hoc* analysis with Dunnett’s *t*-test with Bonferroni adjustment) is applied to compare the differences between the low-altitude group (<1,500 m) and the other groups. IL, interleukin; G-CSF, granulocyte colony-stimulating factor; GM-CSF, granulocyte–macrophage colony-stimulating factor; IFN-γ, interferon γ; IP-10, induced protein 10; MCP-1, monocyte chemoattractant protein 1; MIP-1, macrophage inflammatory protein 1; TNF-α, tumor necrosis factor α; TNF-β, tumor necrosis factor β; VEGF, vascular endothelial growth factor. *P < 0.05, **P < 0.01, ***P < 0.001.

Initial plasma levels of IL-1β, IL-2, IL-4, IL-6, IL-8, IL-10, IL-12, IL-13, IL-15, G-CSF, GM-CSF, IFN-γ, IP-10, MCP-1, MIP-1, TNF-α, TNF-β, and VEGF were detected to compare the cytokines and chemokines of HAdV patients at different altitude areas (**Figure 3**). Plasma levels of IL-1β, IL-2, IL-4, IL-6, IL-8, IL-10, G-CSF, GM-CSF, IFN-γ, IP-10, MCP-1, TNF-α, TNF-β, and VEGF of the ultra-high-altitude group were significantly higher than those of low-altitude group (*P* < 0.05). Meanwhile, a slight rise of IL-2, IL-6, IL-8, IL-10, G-CSF, IP-10, MCP-1, TNF-α, TNF-β, and VEGF was observed in the high-altitude group compared with the low-altitude group (*P* < 0.05). However, there were no differences between the middle-altitude group and the low-altitude group except for IL-6, G-CSF, IP-10, TNF-α, TNF-β, and VEGF (*P* < 0.05).

**Figure 4:**
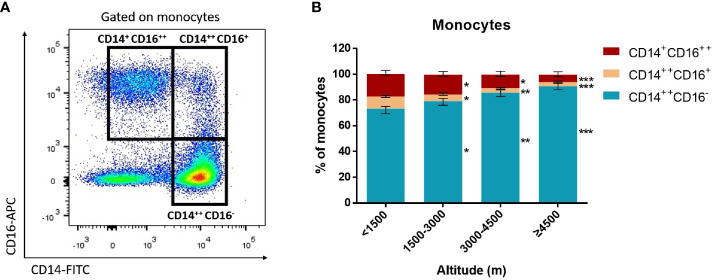
Proportion of monocyte subsets among HAdV infectious patients at different altitudes. Comparison of monocyte subsets among HAdV infectious patients at different altitudes. **(A)** Representative FACS plots of the distributions of monocyte subsets distinguished by CD14 and CD16. **(B)** Comparison of monocyte subset distributions among HAdV infectious patients at different altitudes. The Kruskal–Wallis test (followed by *post-hoc* analysis with Dunnett’s *t*-test with Bonferroni adjustment) is applied to compare the differences between the low-altitude group (<1,500 m) and the other groups. *P < 0.05, **P < 0.01, ***P < 0.001.

**Figure 5:**
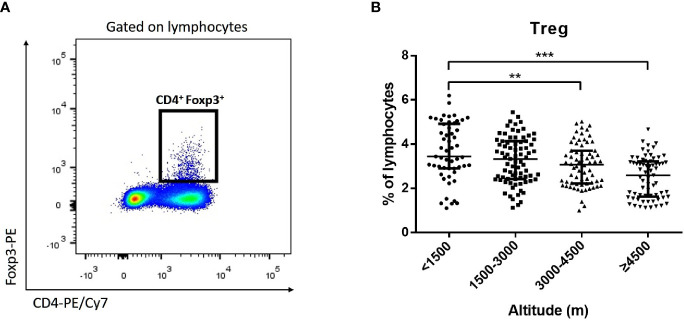
Proportion of Tregs among HAdV infectious patients at different altitudes. Comparison of Tregs among HAdV infectious patients at different altitudes. **(A)** Representative FACS plots of the Tregs distinguished by CD4 and Foxp3. **(B)** Comparison of Tregs among HAdV infectious patients at different altitudes. The Kruskal–Wallis test (followed by *post-hoc* analysis with Dunnett’s *t*-test with Bonferroni adjustment) is applied to compare the differences between the low-altitude group (<1,500 m) and the other groups. **P < 0.01, ***P < 0.001.

Monocytes could be divided into classical monocytes (CD14^++^ CD16^−^), intermediate monocytes (CD14^++^ CD16^+^), and non-classical monocytes (CD14^+^ CD16^++^) according to the expression of CD14 and CD16 (**Figure 4A**) (Cros et al., 2010; Shi and Pamer, 2011). Our results indicated that with the elevation of altitude, the proportion of non-classical monocytes and intermediate monocytes gradually decreased (*P* < 0.05), while classical monocytes increased (*P* < 0.05) (**Figure 4B**). Especially, the median proportion of non-classical monocytes in the ultra-high-altitude group was merely 5.5% (interquartile range, 2.8–8.9). In addition, the expression of CCR2, CX3CR1, and CD86 on the surface of non-classical monocytes in the ultra-high-altitude group was significantly lower than that in the low-altitude group (*P* < 0.05) (**Table 2**). Similarly, we calculated the proportion of Tregs (CD4^+^ Foxp3^+^) of patients at different altitude areas (**Figure 5A**). Compared with the low-altitude group, the proportion of Tregs in the high-altitude group and ultra-high-altitude group was reduced significantly (*P* < 0.05) (**Figure 5B**).

**Table 2:** The receptor expression on the surface of monocyte subsets.

Receptor	Monocytes subsets	Percentage of monocyte subsets (IQR)				*P*-value		
		Low-altitude group (<1,500 m) (*n* = 35)	Medium-altitude group (1,500–3,000 m) (*n* = 109)	High-altitude group (3,000–4,500 m) (*n* = 94)	Ultra-high-altitude group (≥4,500 m) (*n* = 60)	*P*_1_	*P*_2_	*P*_3_
CCR2	CD14^++^ CD16^−^	84.8 (79.2–91.1)	82.6 (76.3–89.4)	81.3 (74.2–88.9)	78.9 (71.5–86.3)	0.78	0.15	0.073
	CD14^++^ CD16^+^	74.1 (69.2–79.8)	72.5 (67.6–78.2)	70.4 (64.3–77.9)	68.9 (63.1–74.8)	>0.99	0.34	0.13
	CD14^+^ CD16^++^	97.4 (95.2–99.7)	94.3 (91.7–97.9)	92.3 (89.4–96.3)	89.2 (86.9–93.4)	0.48	0.11	0.046
CX3CR1	CD14^++^ CD16^−^	63.7 (59.8–68.1)	61.2 (57.3–67.8)	59.6 (54.3–65.9)	56.5 (52.3–61.8)	0.52	0.15	0.084
	CD14^++^ CD16^+^	96.7 (95.2–98.1)	94.4 (89.7–98.2)	91.3 (86.8–95.4)	87.5 (83.2–92.7)	0.35	0.065	0.013
	CD14^+^ CD16^++^	97.5 (96.3–98.8)	92.4 (88.1–96.7)	89.9 (86.5–95.1)	86.1 (82.8–91.2)	0.46	0.17	0.034
CD86	CD14^++^ CD16^−^	17.4 (14.8–21.6)	16.3 (14.2–20.9)	14.7 (11.5–17.9)	11.2 (8.9–16.4)	0.73	0.54	0.18
	CD14^++^ CD16^+^	97.2 (95.7–98.4)	95.8 (93.3–97.5)	93.9 (91.8–96.2)	90.1 (86.5–94.8)	0.87	0.25	0.11
	CD14^+^ CD16^++^	81.5 (77.9–86.3)	78.2 (73.7–84.5)	76.8 (70.2–82.9)	73.3 (68.2–78.7)	0.29	0.045	<0.001

Data are median (IQR), n (%), or n/N (%), where N is the total number of patients with available data. P-values comparing among different altitudes are from χ^2^ test, Fisher’s exact test, or Kruskal–Wallis test (followed by post-hoc analysis with Dunnett’s t-test with Bonferroni adjustment). P_1_ refers to the comparisons between the low-altitude group and medium-altitude group. P_2_ refers to the comparisons between the low-altitude group and high-altitude group. P_3_ refers to the comparisons between the low-altitude group and ultra-high-altitude group.

IQR, interquartile range; CCR2, chemokine C-C receptor 2; CX3CR1, chemokine C-X3-C receptor 1.

-, negative; +, positive; ++, strong positive. The different monocyte subsets are shown in **Figure 4A**.

### Radiologic and Laboratory Findings of HAdV Patients at Different Altitude Areas

Radiology examinations, including chest radiograph and CT scan, were carried out for hospitalized patients and severe patients. Among all of the chest radiographs, 17.2% of HAdV patients had ground-glass opacity and 14.9% had patch shadowing. Compared with the low-altitude group (8.7%), the proportion of patchy shadowing in the high-altitude group (18.3%) and ultra-high-altitude group (20.2%) was significantly higher (*P* < 0.05) (**Supplementary Table 1**). Chest CT was much more sensitive in detecting abnormalities of HAdV infections. The typical manifestation of HAdV infection in chest CT was bilateral multiple lobar and subsegmental areas of consolidation. After a period of treatment, the consolidation would be gradually absorbed and ground-glass opacity would be more apparent (**Supplementary Figure 2**). Different from the results of chest radiographs, the most common abnormality in chest CT was patch shadowing (31.0%), followed by ground-glass opacity (26.1%). Ground-glass opacity (35.6%), patch shadowing (38.8%), vascular enlargement (34.6%), interlobular septal thinning (31.9%), and air bronchogram sign (28.7%) in the ultra-high-altitude group were significantly higher than those in the low-altitude group (*P* < 0.05) (**Supplementary Table 1**).

Despite within the normal range, the white blood cell count of patients was still at a relatively low level (4.9 × 10^9^/L, interquartile range, 3.8–5.8) (**Supplementary Table 2**). Compared with the low-altitude group (5.5 × 10^9^/L, interquartile range, 4.8–6.0), the white blood cell count level of the high-altitude group (4.5 × 10^9^/L, interquartile range, 3.7–5.5) and ultra-high-altitude group (3.9 × 10^9^/L, interquartile range, 2.8–5.3) was significantly lower (*P* < 0.05) (**Supplementary Table 2**). Equally, this feature was also suitable for lymphocyte count and platelet count. Furthermore, the lymphocyte counts of the high-altitude group (1.0 × 10^9^/L, intermediate range, 0.6–1.3) and the ultra-high-altitude group (0.9 × 10^9^/L, intermediate range, 0.5–1.2) were lower beyond the normal range. However, D-dimer, creatine kinase-MB (CK-MB), lactate dehydrogenase (LDH), alanine aminotransferase (ALT), aspartate aminotransferase (AST), and blood urea nitrogen (BUN) were significantly increased in the high-altitude group and the ultra-high-altitude group (*P* < 0.05) (**Supplementary Table 2**).

Subsequently, major laboratory markers were tracked from illness onset (**Supplementary Figure 3**). On the whole, the levels of white blood cell count, lymphocyte count, and platelet count were significantly decreased with the elevation of altitude, while D-dimer, BUN, and creatinine were increased. Although the white blood cell count and lymphocyte count in the low-altitude group reached the bottom at 6 days after disease onset, the decline was more likely to be extended in other groups. Especially, the decline in the ultra-high-altitude group lasted until 18 days and followed by slow recovery. BUN and creatinine levels in the low-altitude group almost did not change during the observation. However, visible variations were obtained in both the middle-altitude group and the high-altitude group. Notably, a rapid rise of BUN and creatinine in the ultra-high-altitude group was continued as long as 18 days after disease onset.

## Discussion

HAdVs, a common cause of acute respiratory diseases, is a double-stranded linear DNA virus with a diameter of 70–90 nm. Life-threatening HAdV pneumonia has been recorded in military employees, AIDS patients, and transplant recipients (Chen et al., 2020). Due to the innate characteristics of DNA, HAdVs are strongly resistant to general environmental changes and are difficult to inactivate. In addition, HAdVs are highly infectious transmitting directly from person to person and indirectly from the surrounding environment to a person (Bremner et al., 2009). A large number of epidemic episodes appeared in the community, demonstrating that proximity was the key factor for outbreaks (Bremner et al., 2009; Greber and Flatt, 2019). Our findings showed that HAdV epidemics might be not as optimistic as we thought in Tibet. During the 2 years of observation, six relatively large-scale HAdV outbreaks were recorded. However, we must emphasize that the HAdV epidemics are able to be effectively controlled and prevented if proper measures are carried out. After outbreaks, under the guidance of experts from the CDC and the General Hospital, the administrative departments quickly took vigorous and multifaceted measures, such as traffic restrictions, cancellation of gatherings, centralized quarantine, strengthening disinfection, and universal symptom survey. Confirmed cases, suspected cases, and close contacts were identified for quarantine or medical observation.

Environmental factors play an important role during the transmission of respiratory viruses. Our results indicated a positive correlation between the number of HAdV infections and altitude. HAdV susceptibility of residents in Tibet was described previously even though similar mutation patterns and closer genetic evolutionary distance were confirmed in the sequences of HAdVs isolated from Lhasa (3,650 m) and Chengdu (520 m) (Wang et al., 2017), which was partly attributed to the dysfunctions of the immune system at high altitude (Rijssenbeek-Nouwens et al., 2012; Boonpiyathad et al., 2020). Humidity was a vital environmental factor related to seasonal variation, especially in plateau climate. Our study found that the number of HAdV infections was negatively correlated with relative humidity, which was consistent with previous studies (Xu et al., 2021). Interestingly, the number of patients had nothing to do with temperature or oxygen content. It is traditionally believed that people were more likely to be infected in winter and spring when the ambient temperature and oxygen content were extremely low (Cao et al., 2014). However, our findings seemed to be discordant with the concepts. First of all, temperature of the plateau climate that consists of the rainy season and the dry season is relatively low all around the year. Therefore, the influence of temperature may be partly comprised of climatic characteristics. Secondly, although oxygen content is lower with the elevation of altitude, plants seem to provide additional oxygen to partly improve the anoxic situation. Thus, oxygen content and altitude have opposite effects on the number of patients. Finally, due to the small number of patients enrolled, statistical errors cannot be neglected.

Because the relationship of altitude and relative humidity with the number of patients has been revealed in our study, reducing the altitude and increasing the humidity may provide effective measures to relieve the symptoms and promote recovery in the process of clinical treatment. Especially for severe patients in high-altitude areas, transferring to a lower altitude may accelerate the cure rate of patients. If patients are not able to be transferred immediately, increasing the relative humidity appropriately in the dormitory and ward, such as the use of a humidifier, seems to help treatment and avoid virus spreading (Zhou et al., 2020). However, the effect of these measures on patients still needs further clinical trials to confirm in the future.

Field investigation found that some HAdV patients, despite only a few, still caused virus spread after discharge or quarantine. Due to the disorder and abnormality of the immune system at high altitude, the course of the disease might be longer than that at low altitude, which was also consistent with previous studies (Murray, 2014; Brakema et al., 2019). On the other hand, a prolonged course of infection might be assumed to result in the changes of the incubation period, serial interval, and PCR-positive duration. Therefore, especially in high-altitude areas, only those who have been confirmed both clinical remission and negative PCR tests are able to be released from the quarantine. However, the real reasons for this phenomenon have not been clearly explained. Further research should be carried out to ensure whether the alteration for natural characteristics of HAdVs or the dysfunctions of the immune system matters in the process of the epidemiological changes along with the altitude.

Similarities of clinical characteristics after HAdV infection at different altitude areas have been noted. In this cohort, most patients presented with fever, dryness, sputum, and fatigue. The proportion of current smoking in the high-altitude group and the ultra-high-altitude group was significantly higher. This situation may indicate that smoking is more likely to cause HAdV infections in high-altitude areas. Previous studies showed that a greater proportion of smoking in high-altitude areas may be partly attributed to social aspects of low education attainment, poverty, and lack of health insurance coverage (Mercado et al., 2021; Reitsma et al., 2021). In the chest CT scans, the main manifestations were patchy shadow and bilateral ground-glass shadow. Laboratory results showed that white blood cell count and lymphocyte of patients at high-altitude areas decreased more significantly. Medical staff should be alert enough to the possibility of co-infection with other microbes for HAdV infections. Overall, the higher the altitude is, the worse the clinical symptoms and laboratory results are. However, we also found that most of the patients (54%) were asymptomatic, which might contribute greatly to the virus epidemics around the camp and hospital. It is strongly recommended that precautions against airborne transmission should be taken, such as fit-tested N95 masks and other personal protective equipment. To prevent the further spread of the disease, the fever and respiratory symptoms of the infected patients should be closely monitored. Once the diagnosis is suspected, the respiratory tract specimens should be tested immediately. Both pathogen detection and exposure history should be taken into consideration for the identification of asymptomatic infection.

The analysis of cytokines and immunocytes in patients with acute respiratory infectious diseases has always been the research hot spot. Early studies had shown that the increase of serum proinflammatory cytokines (such as IL-1β, IL-6, IL-12, IFN-γ, IP-10, and MCP-1) was associated with extensive lung injury in SARS (Wong et al., 2004). SARS-CoV-2 infectious patients also had the promotion of IL-1β, IFN-γ, IP-10, and MCP-1 (Fajgenbaum and June, 2020). Our study found that a variety of cytokines including IL-1β, IL-2, G-CSF, GM-CSF, IFN-γ, IP-10, MCP-1, and TNF-α in the serum of patients at the highland were significantly increased, which might lead to the activated T helper cell-1 (Th1) response. Therefore, cytokine storms were more likely to occur at higher altitude areas. At the same time, the proportion of non-classical monocytes and Tregs, as well as the chemokine receptors expressed on the monocytes, was appropriately decreased in HAdV patients at high altitude. Previous studies had demonstrated that non-classical monocytes and Tregs played a critical role in antivirus and the maintenance of immune balance (Munoz-Rojas and Mathis, 2021; Robinson et al., 2021). Serious conditions and a prolonged course in the high-altitude area might be partly attributed to the decline of these two subsets. Non-classical monocytes play a great role in both antiviral effects. A previous study demonstrated that non-classical monocytes promoted neutrophil adhesion at the endothelial interface *via* the secretion of TNF-α and strengthened lymphocytes to produce a specific antibody for the virus (Chimen et al., 2017). In spite of only a few studies toward intermediate monocytes, this subset has the highest capacity to present antigen to T cells and induce the highest IL-10 and IFN-γ production when the body is infected by pathogens (Zawada et al., 2011; Hussen et al., 2020). Here, the decrease of non-classical monocytes and intermediate monocytes in high-altitude areas may result in a susceptibility to HAdVs. Besides, patients in high-altitude areas showing a higher possibility to suffer from severe pneumonia may be partly attributed to the decrease of these two subsets. However, further studies are needed to describe the pathogenesis and mechanisms of immune disorders. A biopsy study will be the key to understand this phenomenon.

Our study still has some limitations. First of all, HAdVs were not typed when PCR tests were performed on the respiratory specimens. Therefore, we had no idea about the type of HAdVs in our cohort. Considering the various clinical symptoms caused by different types of HAdVs, non-typing might be one of the most important defects in this study. Moreover, considering the limitations of the diagnostic kit, the exact viral load of HAdVs may be unavailable in the current study. Secondly, most patients in our study were primary men age 20–30. We must admit the selection bias that existed in our study. Thirdly, with the limited number of cases, it was inevitable to avoid certain statistical errors for the small number of groups. Larger cohort data would provide more meaningful results to further determine the clinical symptoms, environmental factors, and epidemiological characteristics of patients at different altitude areas. At the same time, the outcome of the statistical test and the *P*-value should be carefully explained. Non-significant *P*-value did not necessarily exclude the differences among groups. Fourth, the epidemiological resources and laboratory test records of some cases were not able to be extracted completely. Some cases were diagnosed in the local outpatient settings, where the information was just briefly documented and incomplete laboratory tests were carried out. Compared with the integrated documents, some of these local outpatient data are missing although great efforts have been made to contact the attending physicians at that time. Last but not least, no doubt, we had missed some asymptomatic or mild patients. The majority of samples were nasopharyngeal swabs, despite few blood and fecal specimens. Blood and fecal specimens were only applied when the oropharyngeal region was injured or the patient cannot tolerate the swab collections. However, we must admit that blood and fecal specimens may result in the missing of low viral load patients because of the relatively low detection rates of these two specimens (Lam et al., 2021). Therefore, our cohort is more likely to represent typical HAdV cases.

## Conclusions

Here, we provide an initial assessment of clinical and epidemiological characteristics of HAdV patients at different altitude areas. Unexpectedly, the number of infected patients was correlated with altitudes and relative humidity rather than temperature and oxygen content. Thus, patients are more likely to benefit from the implementation of transference to a lower altitude or more humid areas. Secondly, the epidemiological characteristics of patients in high-altitude areas are varied from those in low altitude. Patients at high-altitude areas may need longer treatment and quarantine to guarantee a cure and avoid virus shedding. Thirdly, there are significant differences in clinical symptoms and laboratory and imaging findings among HAdV patients in Tibet, where a vast altitude span emerges. Diagnosis of this disease seemed to be complicated by the diversity in symptoms, radiological results, and severity of diseases. During the clinical works, much more attention should be paid to observe changes in the conditions of patients to help them recover, especially for those in high-altitude areas.

## Data Availability Statement

The original contributions presented in the study are included in the article/**Supplementary Material**. Further inquiries can be directed to the corresponding authors.

## Ethics Statement

The studies involving human participants were reviewed and approved by the Research Ethics Commission of the General Hospital. Written informed consent for participation was not required for this study in accordance with the national legislation and the institutional requirements.

## Author Contributions

BW and WK were responsible for the study concept, designed the study, and took responsibility for the integrity of the data and the accuracy of the data analysis. BW, MP, and WK drafted the paper. BW, LY, GL, CY, XZ, QW, QH, and JY did the analysis and gave final approval for the version to be published. BW, LY, GL, CY, CL, KD, KP, and JY collected the data. All authors agree to be accountable for all aspects of the work in ensuring that questions related to the accuracy or integrity of any part of the work are appropriately investigated and resolved. All authors contributed to the article and approved the submitted version.

## Funding

This study was supported by grants from the Major Program of National Natural Science Foundation of China (2017ZX10202101-004-005) and the Major Program of National Natural Science Foundation of China (2017ZX10202101-003-007).

## Conflict of Interest

The authors declare that the research was conducted in the absence of any commercial or financial relationships that could be construed as a potential conflict of interest.

## Publisher’s Note

All claims expressed in this article are solely those of the authors and do not necessarily represent those of their affiliated organizations, or those of the publisher, the editors and the reviewers. Any product that may be evaluated in this article, or claim that may be made by its manufacturer, is not guaranteed or endorsed by the publisher.
