# Supplementary material for: Clinical and Immunological Characteristics of Patients With Adenovirus Infection at Different Altitude Areas in Tibet, China
Source: Front Cell Infect Microbiol. 2021 Oct 13;11:739429. doi: 10.3389/fcimb.2021.739429 (PMC8548869; doi:10.3389/fcimb.2021.739429)
Supplement: Supplementary file 1 [file DataSheet_1.docx]

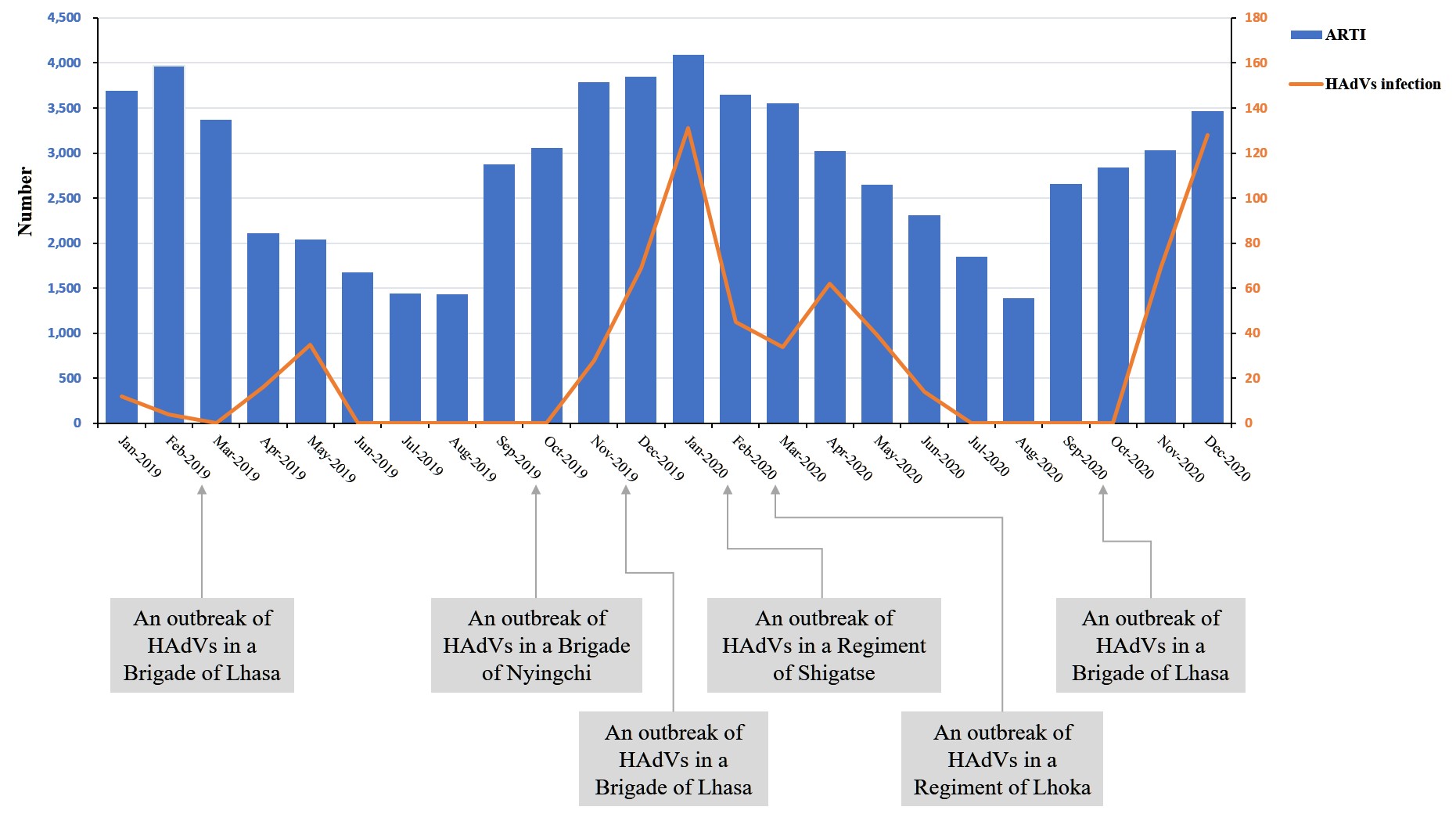


**Supplementary Figure 1** Epidemic curves of respiratory disease and HAdVs infection in Tibet

The number of confirmed cases of respiratory disease (blue) and HAdVs infection (orange) are plotted according to the month of diagnosis. The x-axis of the graph (months from January 2019 to December 2020) is also used as the timeline of epidemic 1. The number of patients with respiratory diseases is shown by blue bar, while the number of patients with adenovirus infection is shown by orange line. The outbreaks of HAdVs in Tibet are shown in grey box. ARTI, acute respiratory tract infections; HAdVs, human adenovirus.

| **Supplementary Table 1** Radiographic findings of study patients | | | | | | | | | |
| --- | --- | --- | --- | --- | --- | --- | --- | --- | --- |
|  | Number (%) | | | | |  | *P* value | | |
|  | Total (n=686) | Low-altitude group (<1500m) (n=62) | Medium-altitude group (1500-3000m) (n=206) | High-altitude group (3000-4500m) (n=230) | Ultra-high-altitude group (≥4500m) (n=188) |  | *P*_1_ | *P*_2_ | *P*_3_ |
| **Abnormalities on chest radiograph** |  |  |  |  |  |  |  |  |  |
| Ground-glass opacity | 118 (17.2) | 6 (9.7) | 29 (14.1) | 46 (20.0) | 37 (19.7) |  | 0.52 | 0.063 | 0.082 |
| Patchy shadowing | 102 (14.9) | 4 (6.5) | 18 (8.7) | 42 (18.3) | 38 (20.2) |  | 0.79 | 0.029 | 0.011 |
| Interstitial abnormalities | 22 (3.2) | 1 (1.6) | 5 (2.4) | 9 (3.9) | 7 (3.7) |  | >0.99 | 0.69 | 0.68 |
| **Abnormalities on chest CT** |  |  |  |  |  |  |  |  |  |
| Ground-glass opacity | 179 (26.1) | 8 (12.9) | 38 (18.4) | 66 (28.7) | 67 (35.6) |  | 0.34 | 0.013 | <0.001 |
| Patchy shadowing | 213 (31.0) | 10 (16.1) | 44 (21.4) | 86 (37.4) | 73 (38.8) |  | 0.47 | <0.001 | <0.001 |
| Interstitial abnormalities | 67 (9.8) | 4 (6.5) | 17 (8.3) | 26 (11.3) | 20 (10.6) |  | 0.79 | 0.093 | 0.46 |
| Vascular enlargement | 186 (27.1) | 10 (16.1) | 40 (19.4) | 71 (30.9) | 65 (34.6) |  | 0.71 | 0.025 | 0.006 |
| Interlobular septal thickening | 164 (23.9) | 10 (16.1) | 41 (19.9) | 53 (23.0) | 60 (31.9) |  | 0.58 | 0.3 | 0.022 |
| Air bronchogram sign | 157 (22.9) | 9 (14.5) | 35 (17.0) | 59 (25.7) | 54 (28.7) |  | 0.7 | 0.089 | 0.028 |
| Discrete pulmonary nodules | 43 (6.3) | 5 (8.1) | 11 (5.3) | 16 (7.0) | 11 (5.9) |  | 0.54 | 0.78 | 0.55 |
| Bronchus deformation | 39 (5.7) | 4 (6.5) | 11 (5.3) | 12 (5.2) | 12 (6.4) |  | 0.76 | 0.75 | >0.99 |
| Pleural effusion | 6 (0.9) | 0 | 1 (0.5) | 2 (0.9) | 3 (1.6) |  | >0.99 | >0.99 | >0.99 |

Data are median (IQR), n (%), or n/N (%), where N is the total number of patients with available data. *P* values comparing among different altitudes are from χ^2^ test, Fisher's exact test, or Kruskal-Wallis test (followed by post hoc analysis with Dunnet-*t* test with Bonferroni adjustment). *P*_1_ refers to the comparisons between the Low-altitude group and Medium-altitude group. *P*_2_ refers to the comparisons between the Low-altitude group and High-altitude group. *P*_3_ refers to the comparisons between the Low-altitude group and Ultra-high-altitude group. IQR, interquartile range; CT: computed tomography.


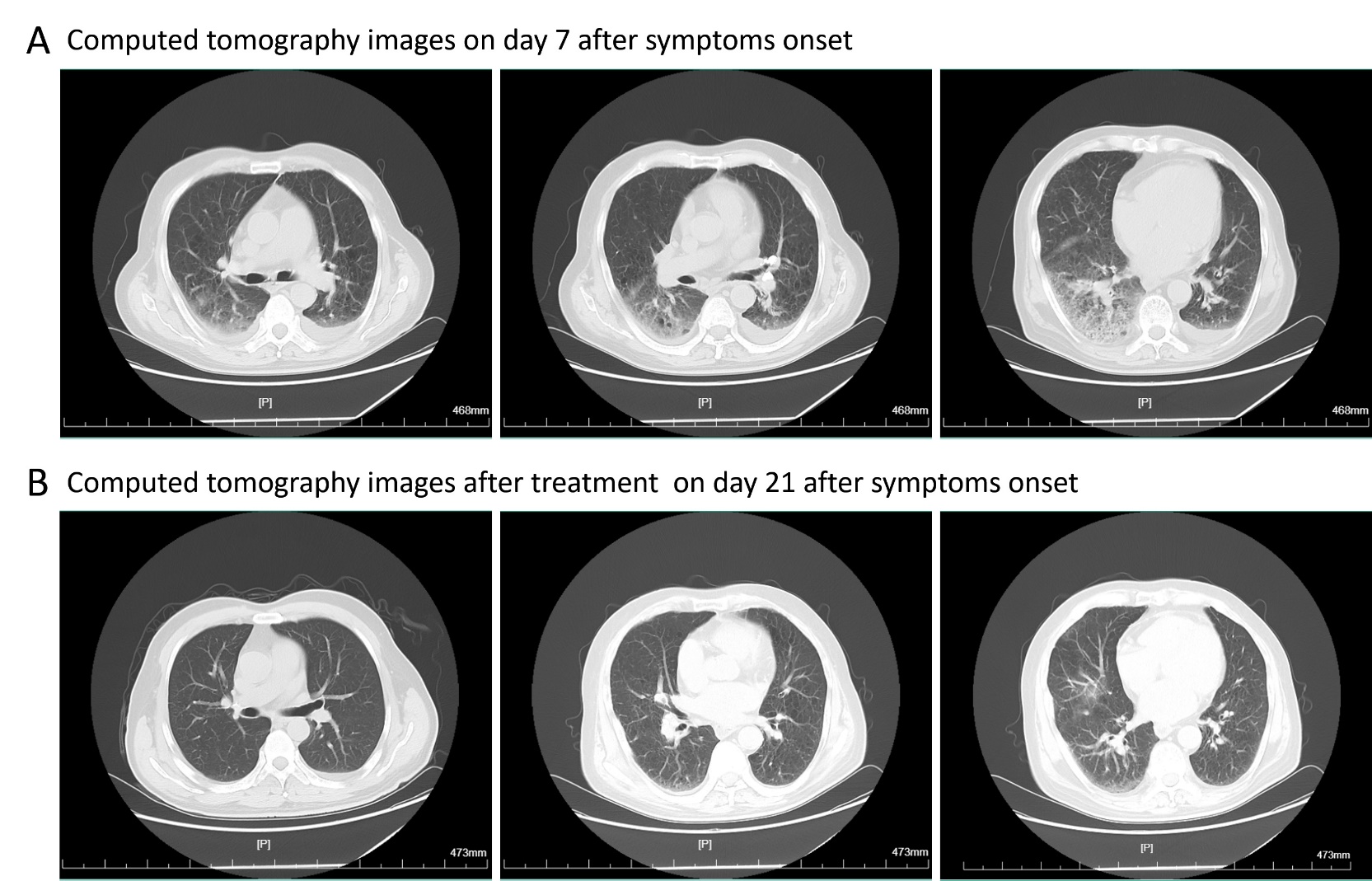


**Supplementary Figure 2** Chest computed tomographic images of a 35 year-old patient infected with HAdVs

A, Multiple consolidation and ground-glass opacity are shown in both lungs on the day 5 after the onset of symptoms. B, Gradually absorption of bilateral consolidation and existence of ground-glass shadow are shown after treatment on the day 21 after the onset of symptoms.

| **Supplementary Table 2** Laboratory findings of study patients | | | | | | | | | | |
| --- | --- | --- | --- | --- | --- | --- | --- | --- | --- | --- |
|  | Normal range | Medium (IQR) | | | | |  | *P* value | | |
|  |  | Total (n=686) | Low-altitude group (<1500m) (n=62) | Medium-altitude group (1500-3000m) (n=206) | High-altitude group (3000-4500m) (n=230) | Ultra-high-altitude group (≥4500m) (n=188) |  | *P*_1_ | *P*_2_ | *P*_3_ |
| White blood cell count, ×10^9^/L | 3.5-9.5 | 4.9 (3.8-5.8) | 5.5 (4.8-6.0) | 5.1 (4.6-5.9) | 4.5 (3.7-5.5) | 3.9 (2.8-5.3) |  | 0.78 | 0.029 | <0.001 |
| Neutrophil count, ×10^9^/L | 1.8-6.3 | 3.3 (2.1-5.0) | 3.3 (2.3-5.2) | 3.4 (2.4-5.4) | 3.3 (2.0-5.1) | 3.2 (2.0-4.5) |  | >0.99 | 0.64 | 0.072 |
| Lymphocyte count, ×10^9^/L | 1.1-3.2 | 1.2 (0.8-1.8) | 1.5 (1.1-1.9) | 1.3 (0.9-1.8) | 1.0 (0.6-1.3) | 0.9 (0.5-1.2) |  | 0.043 | <0.001 | <0.001 |
| Monocyte count, ×10^9^/L | 0.1-0.6 | 0.4 (0.2-0.7) | 0.3 (0.1-0.4) | 0.4 (0.3-0.6) | 0.4 (0.2-0.8) | 0.4 (0.2-0.7) |  | 0.94 | 0.73 | 0.75 |
| Platelet count, ×10^9^/L | 115-350 | 220 (164-283) | 263 (215-320) | 241 (176-308) | 207 (149-272) | 178 (109-255) |  | 0.039 | <0.001 | <0.001 |
| Prothrombin time, s | 9.4-12.5 | 11.6 (10.9-12.4) | 11.4 (10.9-11.9) | 11.8 (11.0-12.5) | 11.3 (10.7-11.8) | 11.6 (11.0-12.1) |  | 0.18 | >0.99 | >0.99 |
| APTT, s | 25.1-36.5 | 31.5 (29.4-33.6) | 30.6 (28.3-31.9) | 31.3 (28.9-33.7) | 33.0 (30.6-35.3) | 35.8 (32.4-38.7) |  | >0.99 | 0.17 | 0.015 |
| D-dimer, mg/L | 0-500 | 364 (215-526) | 207 (144-273) | 272 (184-375) | 384 (269-517) | 439 (308-557) |  | 0.047 | <0.001 | <0.001 |
| CK, U/L | <171 | 98 (56-141) | 72 (43-96) | 85 (58-107) | 101 (68-136) | 128 (97-163) |  | 0.76 | 0.11 | 0.026 |
| CK–MB, U/L | <25 | 14 (9-18) | 8 (5-12) | 12 (9-15) | 17 (13-23) | 20 (15-24) |  | 0.014 | <0.001 | <0.001 |
| LDH, U/L | 125-243 | 258 (172-340) | 146 (109-197) | 202 (161-253) | 279 (225-331) | 336 (284-379) |  | 0.039 | <0.001 | <0.001 |
| ALT, U/L | 9-50 | 30 (21-38) | 18 (14-23) | 23 (19-26) | 31 (24-38) | 35 (30-41) |  | 0.19 | 0.032 | <0.001 |
| AST, U/L | 10-45 | 28 (20-36) | 19 (13-24) | 24 (20-27) ^*^ | 31 (23-37) ^△^ | 34 (26-41) |  | 0.27 | <0.001 | <0.001 |
| CRP, 10mg/L | <10 | 4 (2-6) | 3 (1-4) | 3 (1-5) | 4 (2-7) | 4 (2-7) |  | 0.74 | >0.99 | >0.99 |
| TB, mmol/L | 5-21 | 9.7 (7.5-12.3) | 8.6 (6.7-10.6) | 9.3 (7.5-11.0) | 10.3 (8.4-12.3) | 13.6 (12.0-15.8) |  | 0.89 | 0.12 | 0.013 |
| BUN, mmol/L | 2.8-7.6 | 4.8 (4.4-7.3) | 4.2 (3.6-4.7) | 4.7 (4.0-5.9) | 5.6 (5.0-6.1) | 6.3 (5.8-6.9) |  | 0.023 | <0.001 | <0.001 |
| Creatinine, μmol/L | 60-104 | 76 (65-93) | 71 (63-81) | 74 (69-83) | 78 (71-88) | 89 (80-96) |  | 0.87 | 0.16 | 0.016 |

Data are median (IQR), n (%), or n/N (%), where N is the total number of patients with available data. *P* values comparing among different altitude areas are from χ^2^ test, Fisher's exact test, or Kruskal-Wallis test (followed by post hoc analysis with Dunnet-*t* test with Bonferroni adjustment). *P*_1_ refers to the comparisons between the Low-altitude group and Medium-altitude group. *P*_2_ refers to the comparisons between the Low-altitude group and High-altitude group. *P*_3_ refers to the comparisons between the Low-altitude group and Ultra-high-altitude group. ^*^Data regarding the CRP of Medium-altitude group were missing for 18 patients; ^△^Data regarding the CRP of High-altitude group were missing for 21 patients. IQR, interquartile range. APTT, activated partial thromboplastin time; CK, creatine kinase; CK-MB, creatine kinase-MB; LDH, lactic dehydrogenase; ALT, alanine transaminase; AST, aspartate aminotransferase; CRP, c reactive protein; TB, total bilirubin; BUN, blood urea nitrogen.


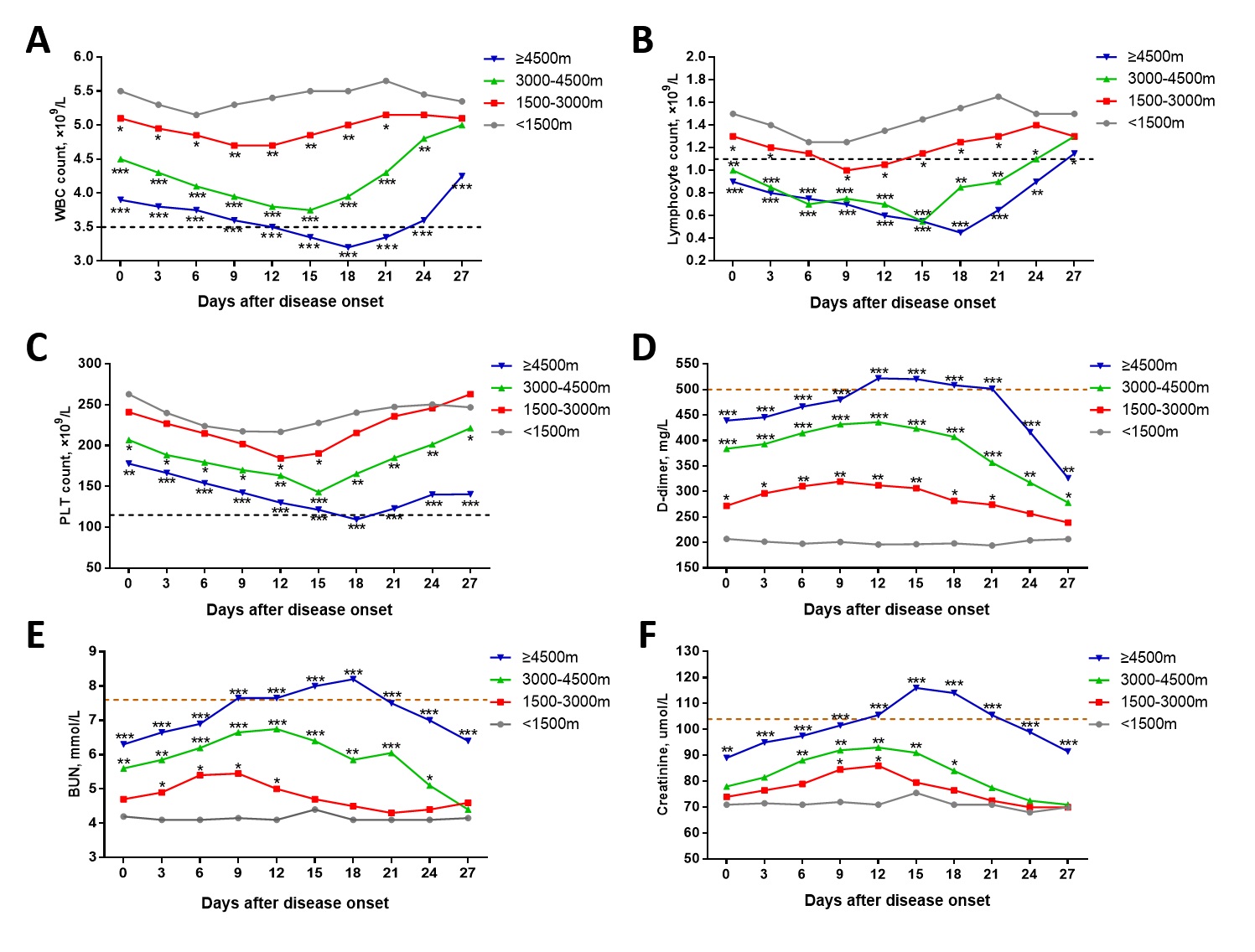


**Supplementary Figure 3** Dynamic profile of laboratory markers from illness onset in patients infected with HAdVs

Figure shows the time changes of WBC count (A), Lymphocyte (B), PLT (C), D-dimer (D), BUN (E) and Creatinine (F). Timeline charts show the laboratory parameters of patients infected with HAdVs every two days from the illness onset. The black dotted line represents the lower normal limit of each parameter, and the brown dotted line represents the upper normal limit of each parameter. Kruskal-Wallis test (followed by post hoc analysis with Dunnet-*t* test with Bonferroni adjustment) is applied to compare the differences between low-altitude group (<1500m) and other groups. HAdVs, human adenovirus; WBC, white blood cell; PLT, platelets; BUN, blood urea nitrogen; ^＊^*P*<0.05, ^＊＊^*P*<0.01, ^＊＊＊^*P*<0.001.
